# Novel Approach to Tooth Chemistry: Quantification of Human Enamel Apatite in Context for New Biomaterials and Nanomaterials Development

**DOI:** 10.3390/ijms22010279

**Published:** 2020-12-29

**Authors:** Andrzej Kuczumow, Renata Chałas, Jakub Nowak, Wojciech Smułek, Maciej Jarzębski

**Affiliations:** 1 ComerLab Dorota Nowak Radawiec Duży 196, 21-030 Motycz, Poland; andrzej.kuczumow@gmail.com (A.K.); kubit75@gmail.com (J.N.); 2 Department of Oral Medicine, Medical University of Lublin, Chodźki 6, 20-093 Lublin, Poland; renata.chalas@umlub.pl; 3 Institute of Chemical Technology and Engineering, Poznan University of Technology, Berdychowo 4, 60-695 Poznan, Poland; wojciech.smulek@put.poznan.pl; 4 Department of Physics and Biophysics, Poznan University of Life Sciences, Wojska Polskiego 38/42, 60-637 Poznan, Poland

**Keywords:** teeth enamel, apatites, electron microscopy, X-ray diffraction, quantification, energies of crystal transformation

## Abstract

A series of linear profiles of the elements of the enamel in human molar teeth were made with the use of an electron microprobe and a Raman microscope. It is postulated that the enamel can be treated as the superposition of variable “overbuilt” enamel on the stable “core” enamel at the macro-, micro- and nanoscale level. The excessive values characterize the “overbuilt enamel”. All the profiles of excessive parameters along the enamel thickness from the enamel surface to the dentin enamel junction (DEJ) can be approximated very precisely with the use of exponential functions, where Ca, P, Cl and F spatial profiles are decaying while Mg, Na, K and CO_3_^2−^ ones are growing distributions. The “overbuilt” apatite formed on the boundary with DEJ, enriched in Na, Mg, OH and carbonates, reacts continuously with Ca, Cl and F, passing into an acid-resistant form of the “overbuilt” enamel. The apparent phases arriving in boundary regions of the “overbuilt enamel” were proposed. Microdiffraction measurements reveal relative variation of energy levels during enamel transformations. Our investigations are the milestones for a further new class of biomaterial and nanomaterial development for biomedical applications.

## 1. Introduction

There is a need to develop new materials for dentistry, especially dedicated to fillings and prosthesis purposes. Recently, a number of studies were focused on the possible application of various nanomaterials, such as titanium alloys [1] or hydroxyapatite nanocomposites [2], for dentistry application and bone and tissue restoration. Nanocomposites showed benefits for dental applications such as antibacterial effects, Ca and PO_4_^3−^ ion release or prevention from demineralization and hardness lost [3]. Nevertheless, for further nanomaterials’ development, elemental knowledge of the chemical profiles of different elements in teeth is necessary.

Only a few biomaterials have been studied in a comparably extensive way as the tooth enamel, especially the human one [4,5,6,7]. This apparently simple material is difficult to fully understand [8]. Although the vast majority of tissue belongs to the inorganic phase of modified carbonated hydroxyapatite [9,10,11], with a small (~2%) but essential [12] admixture of organic material and similar contents of water, the important details of the material structure remain obscure. There have been numerous studies on the chemical content of the enamel, both at macroscale [13,14], leading to the total determination of averaged concentrations (and mechanical parameters) and at the semimicro- [15] and microscale [16,17], showing the spatial distributions of elements [18,19,20,21,22,23]. The investigations concerned main, minor and trace [24,25,26] elements, even in the aspect of the diet [27]. The observations of role of some minor but important elements such as Mg, Sr, Na, Cl and F in the apatite matrix were conducted [28,29,30,31,32,33,34,35,36,37]. Such studies were sometimes supported by acid etching at the semimicro scale, treated as proof for the regular change in the chemical resistance of the tooth [38]. The scientific papers also involved the problem of the element uptake inside enamel and, in a more general sense, inside tooth [39]. In parallel, significant studies on the detailed crystallographic determination of the structure were undertaken [40,41,42] and aimed at coupling small changes in the structure with ion substitutions in the lattice, with the possibility of determining variable density. Since the chemical features of enamel surely influence the mechanical characteristics of the tissue, the parallel mechanical studies of enamel were also extensive [43,44,45,46]. Further progress in the instrumentation, especially construction of indentation attachments to AFM led to the mechanical studies at microscale [47]. Ultimately, for humans, as such, the mechanical characteristics of enamel are more important than the kind of chemical material constituting the tooth [48,49,50].

The studies were widened on animal and fossil [51] systems, both since sometimes it is easier to perform such experiments, and for the reason that some animal-based results are instructive in a more general sense [52,53,54]. In parallel, studies on the mimetic syntheses of materials resembling enamel were initiated [55,56,57,58,59,60] and they were supported by rigorous estimation of the enamel’s composition and structure [61].

Meantime, some interesting new information arrived, concerning essentially typical geological minerals, but with connotations towards some chemical aspects of biological apatites. The interrelationships between fluor-, chlor- and hydroxyapatites were shown [62,63,64]. The ranges of existence of particular apatites were determined and conditions of their transformations were shown, dependent on applied pressure and temperature and availability of relevant ions.

Still, even with numerous studies and reviews concerning enamel, the exhaustive phenomenological estimation of enamel variable elemental profiles and structural connections between chemical elements is missing. However, the first step for making such a model was made a long time ago [65]. For some reason, this course of reasoning was nearly discontinued, with small exceptions [66,67]. This study intends to fulfill this drawback, because the authors are convinced that the spatial distributions of the elements tell something very important and, if carefully considered, give insight into the changes in the composition and structure of enamel and, moreover, in the energetics of enamel (for the first time). In summary, it should bring progress in proper preparation of future materials substituting enamel, which ought to be smoothly variable. This contribution is a continuation of our earlier paper [68].

## 2. Results

### Chemical and Mechanical Investigations

The linear scans were made in the direction of the enamel surface or DEJ on lingual surface, following positions indicated in papers [43,69]. No scan of any chemical element showed a constant level of concentration. We always observed regular either systematically decreasing concentrations, as for Ca, P, Cl and F, or constantly increasing values for Na, K, Mg and CO_3_^2−^. For that reason, it was possible to establish some core values, above which the concentrations grew either in the direction of the tooth surface or towards the DEJ. The data estimated as core values are cited in Table 1, where chemical results are our own. The core concentrations are the minimal concentrations met at any point of the sample. This set of parameters is not observed as a whole in any real location of the sample. The set represents the hypothetical “core enamel”. We supplemented it with core values of other essential parameters (hardness, elasticity modulus [44,45], density [46]), adapted from the data found in literature. Since the absolute data are inherently connected with the individual samples and measurement points, all the data were transformed to the relative values, i.e., compared with maximum values. The relative amounts are given in the third row of the table. In our opinion, such relative values visualize in a much better way the potential universal trends. The data in Table 1 allow for estimating the amount of the “overbuilt phase” as, in approximation, 6.5% of the whole enamel mass. It means that real chemical reactions cover 1/15 of the total enamel mass and the rest of the enamel can be considered as an apparently stable and passive structure.

**Figure 1:**
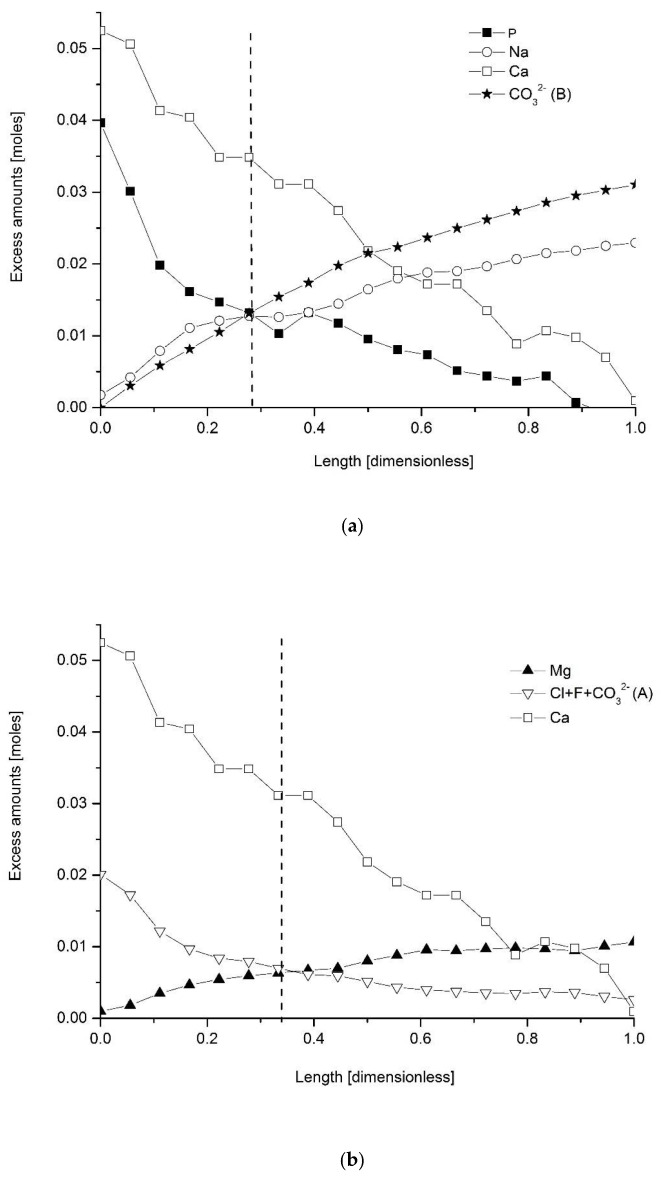
The concentration spatial profiles of (**a**) the excessive content of P, Ca, Na and CO_3_^2−^ (B) as calculated in excessive molar values (see text) against the dimensionless distance, calculated from the enamel surface (left side, position 0) to DEJ (right side of the figure, position 1); (**b**) the same for the excessive amounts of Ca, Mg and Cl + F + CO_3_^2−^ (A), with missing profile of OH^−^ ions. Dotted lines show the intersection points of curves, roughly in the same location. Capital A and B letters mean substitutions for CO_3_^2−^ ions.

Figure 1 shows the excessive amounts of detected elements/ions. Here, “excessive” means the amount over the “core” values summarized in Table 1. It is clear that the excessive values of Ca, P and Cl drop, starting from the enamel surface to the bulk regions, while content of Na and Mg increases, as well as CO_3_^2−^. Concentration values are recalculated to the atomic (molar for CO_3_^2−^) amounts per 100 g of the whole enamel sample for better observation of stoichiometric relationships in the “excessive” mass of the enamel. It is important since, from the assumption, all reactions occur only in “overbuilt enamel”. The results are split in two parts: Figure 1a shows the decreasing streams of Ca and P ions and the increasing ones of Na and CO_3_^2−^ (substitution B). Figure 1b shows the decreasing streams of Ca and Cl + F + CO_3_^2−^ (substitution A) and an increasing stream of Mg ions, with the missing stream of OH^−^ ions (due to inability of their simple quantification). The grouping is done according to the exchange mechanisms which will be presented in Equations (3) and (4) further in the text. An extremely important indication can be drawn from the intersection points for the curves presented in the figures—the points occur at approximately the same location for both reaction mechanisms. Ca curves were not taken into account here since Ca ions participate in both mechanisms.

**Figure 2:**
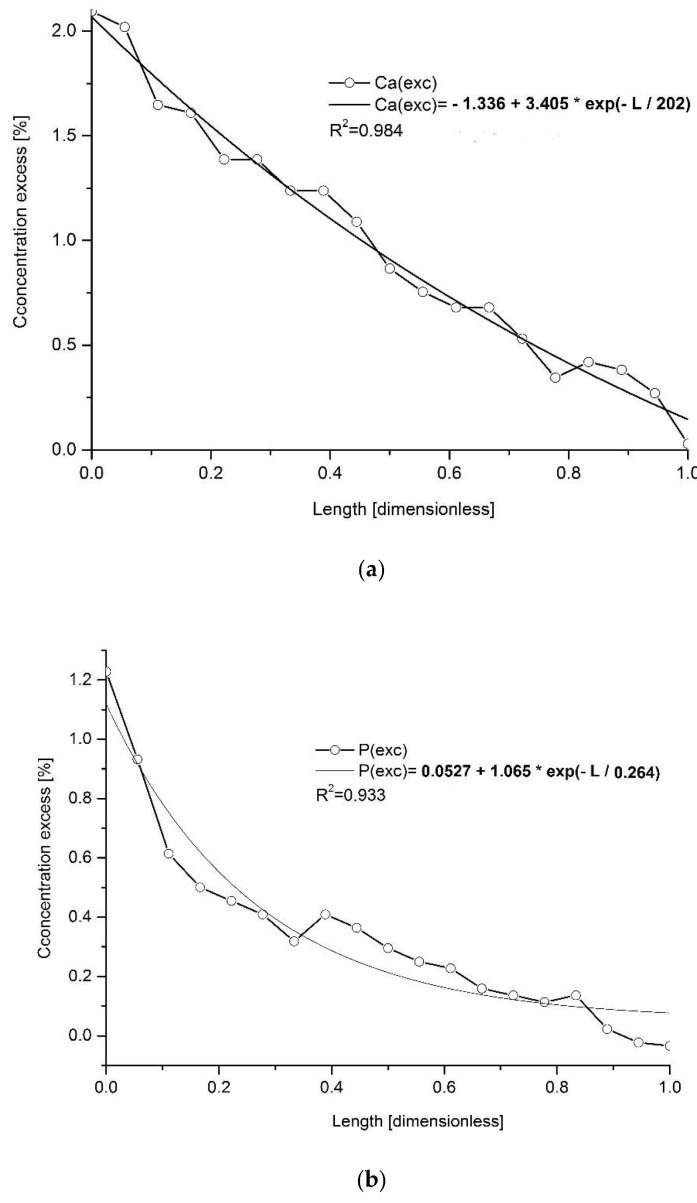

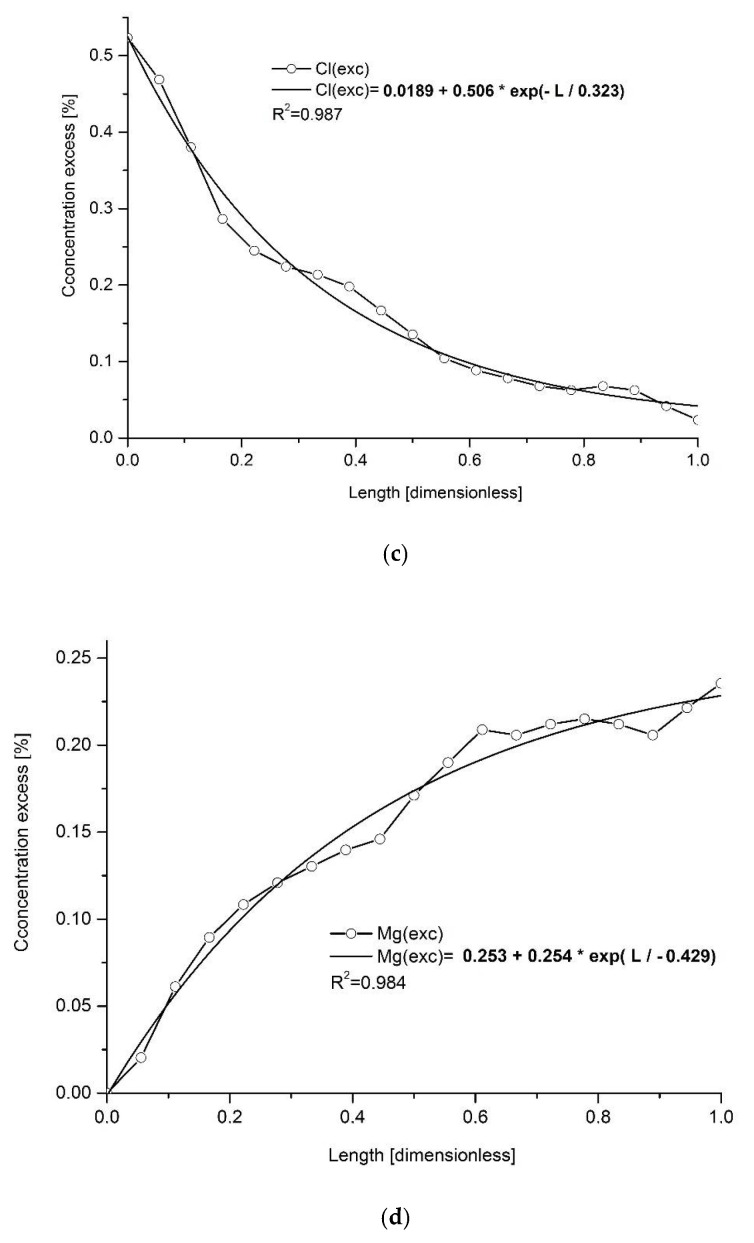

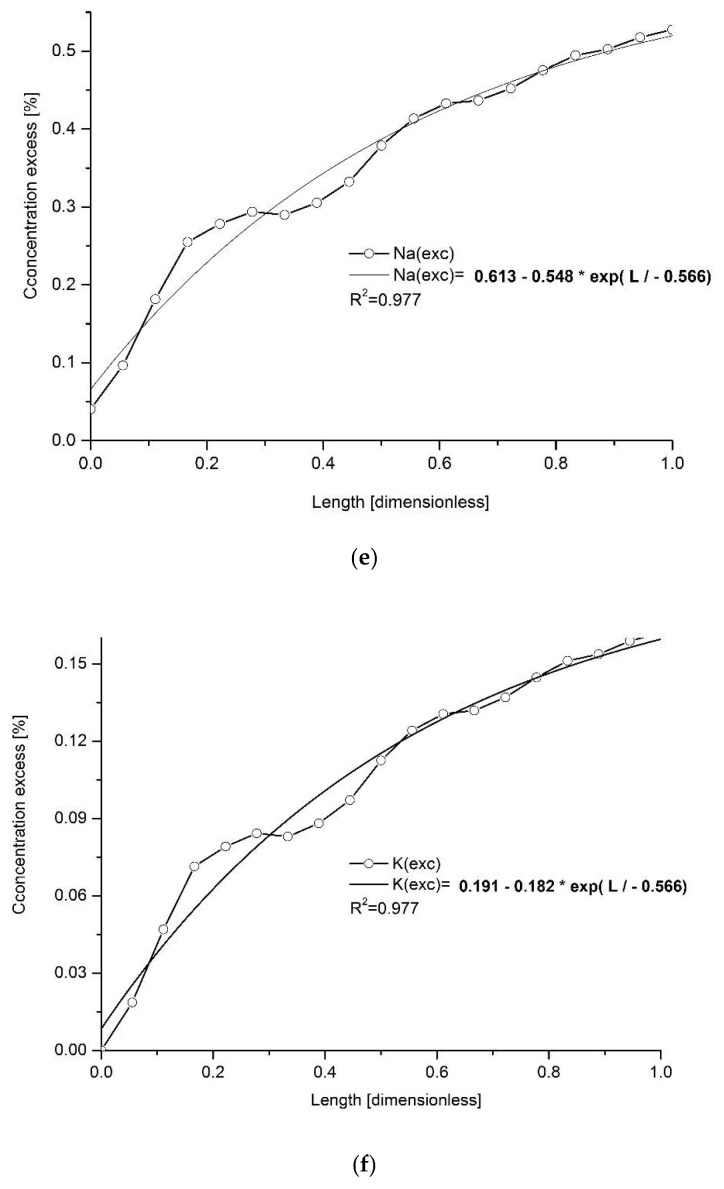

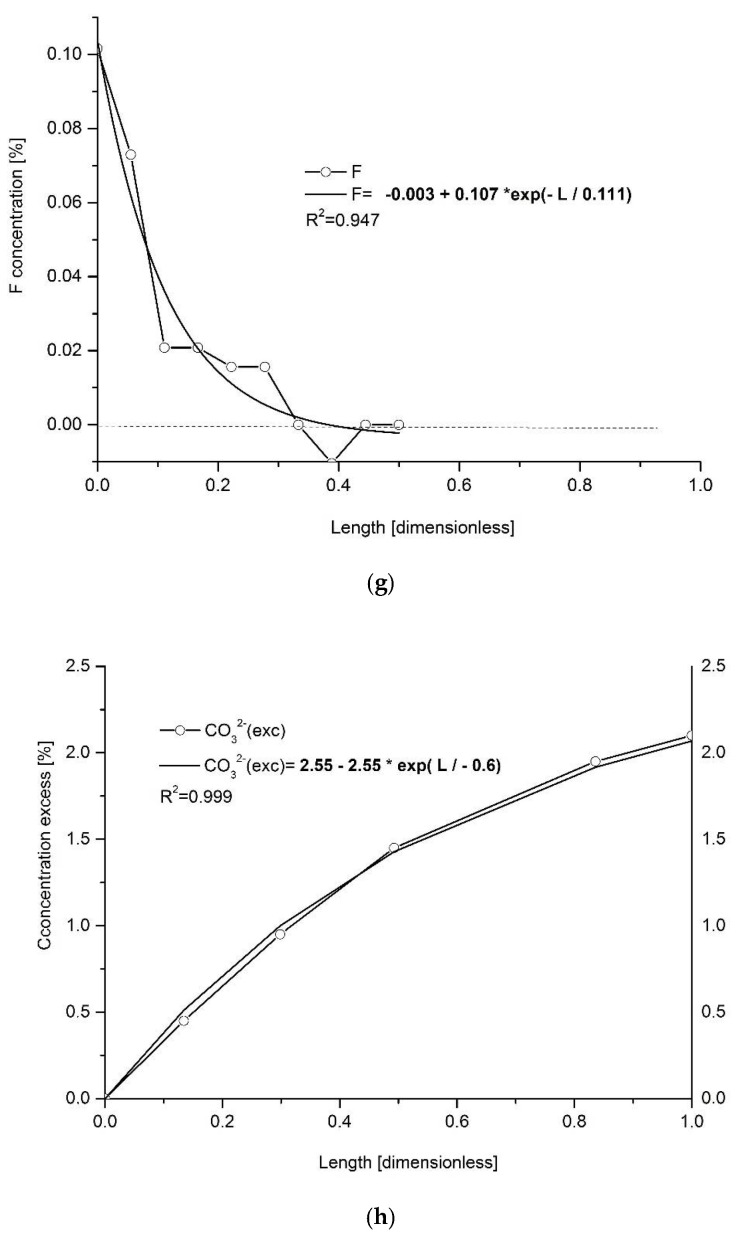

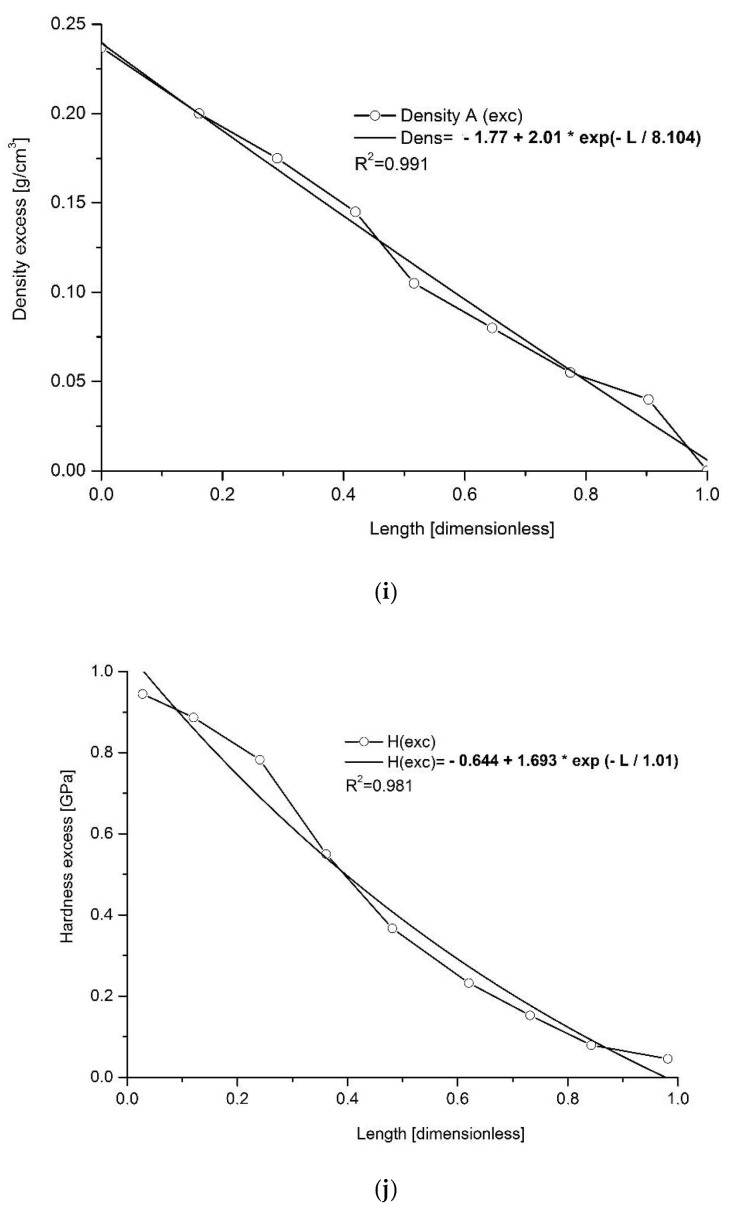
Approximation of the excessive chemical spatial profiles along the human molar enamel with exponential functions for (**a**) Ca; (**b**) P; (**c**) Cl; (**d**) Mg; (**e**) Na; (**f**) K; (**g**) F; (**h**) CO_3_^2−^, all expressed in weight per cents and for (**i**) density and (**j**) hardness excesses—adopted from [44,46], respectively.

All the elemental and molecular excesses, expressed this time as weight percent concentrations, were approximated with the exponential functions, and this approximation worked extremely well (Figure 2). Similarly, some other parameters related to the samples such as density (Figure 2i) and hardness (Figure 2j could be approximated in the same way.

**Figure 3:**
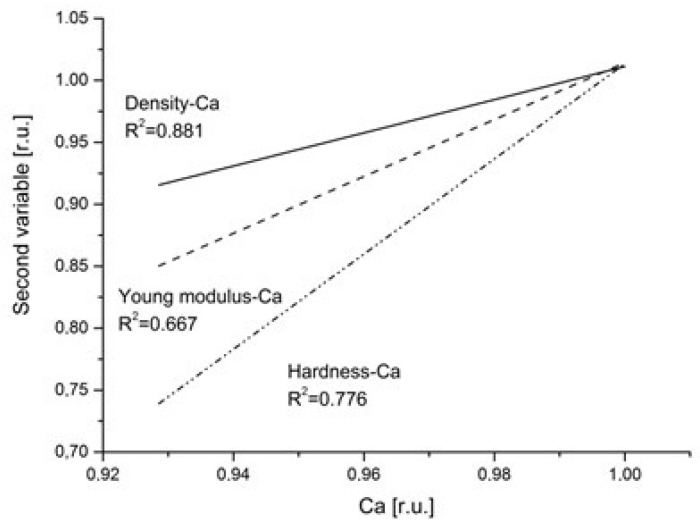
Comparison of the linear fits to the correlation relationships between relative values of density (adopted from [46]) and Ca (solid line); Young modulus and Ca (dotted line); hardness and Ca (dot-drop line)—adopted from [44,45]. Correlation coefficients are cited.

It is important to see how the values of Ca concentration, density and mechanical parameters such as hardness and Young modulus (adapted from positions [43,44,46,70]), expressed in relative values, correlate with one another. We selected approximation to the linear correlation of variables mentioned against the Ca values. Such an approach is illustrated in Figure 3. Only the main element Ca is selected here since it should be mainly responsible for the total structure of the matrix. The results are, in some sense, trivial, e.g., the drop in the density strictly follows that in the Ca content and the proportion of the drop is strictly the same. The correlation coefficient shows that the density is in the strictest way coupled with Ca contents. However, the relative drop in the hardness follows the drop in Ca relative content; the proportion is not 1:1, but the hardness drops down much quicker. The Young modulus drop is moderate in relative units, somewhere between the drop in Ca content and in hardness. The data given in Figure 3 concern the whole enamel and not the base or overbuilt part. It obviously testifies that decalcification is devastating for hardness at first.

**Figure 4:**
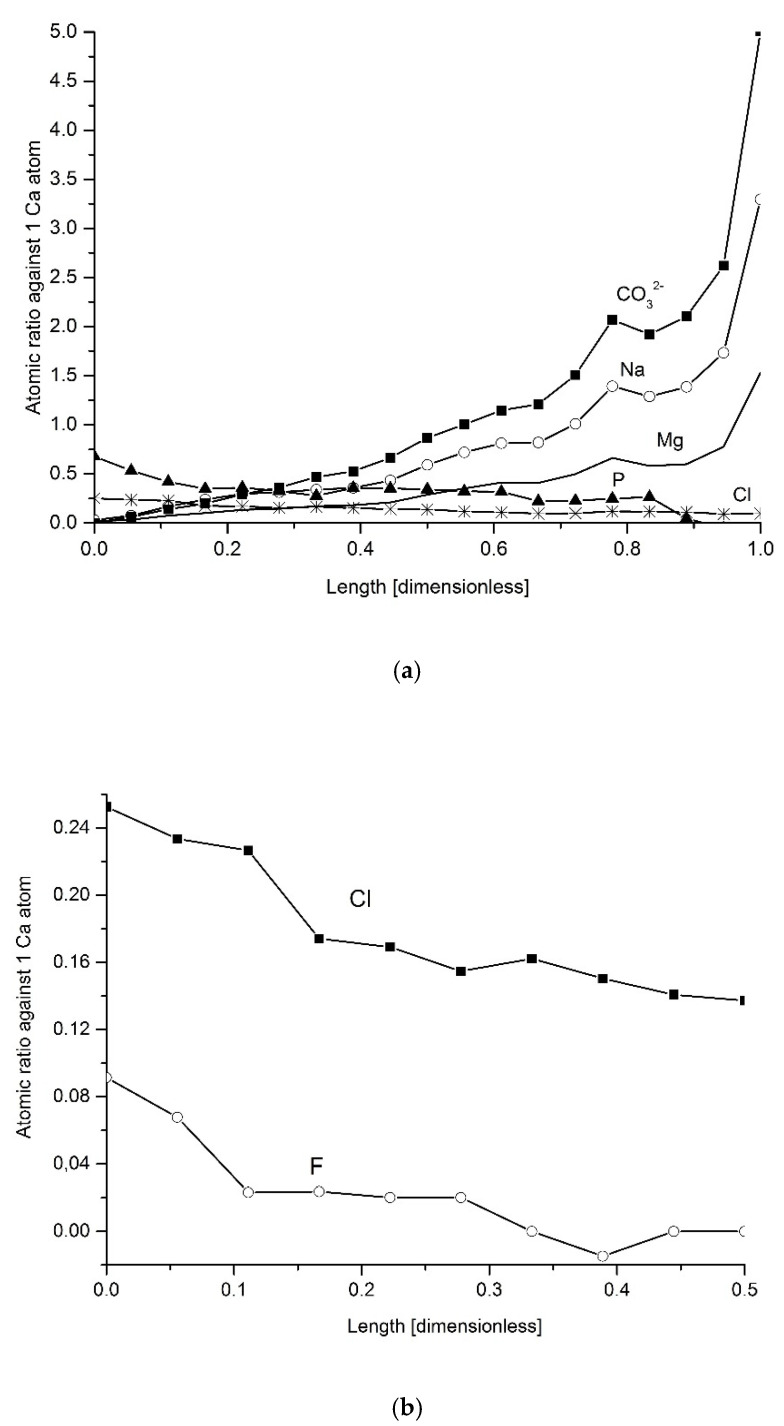

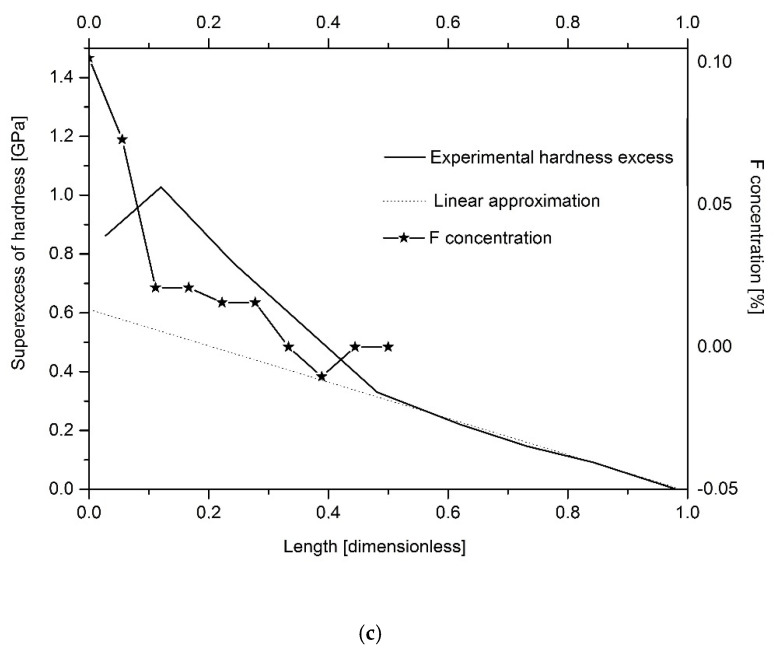
(**a**) Profiles of the atomic relations occurring in the “overbuilt” enamel; the atomic ratios are recalculated per 1 Ca atom for: P—solid line with full triangles; Na—solid with open circles; Mg—solid; Cl—solid with stars; CO_3_^2−^—solid with full squares; (**b**) the same for fluoride, compared with chloride, on a shortened distance corresponding to locations where F is easily detected; (**c**) the comparison of the absolute concentration of F (solid line with stars) with the additional excess of hardness value (solid line) close to the boundary with air.

One can return once more to the so-called “overbuilt enamel”. After recalculation of the “excessive” concentrations to the moles per 100 g sample, we compared the amounts of different atoms and chemical groups per one atom of “excessive” Ca. It is shown in Figure 4. It is surprising, as the relation of P/Ca changes, somewhat, with the growing deficit of P (but we must remember that the majority of both main elements are involved in the “core enamel” and the changes in the “overbuilt enamel” have not so much meaning per total). Surprisingly small is the change in the Cl profile. Still, there is a drop in the Cl participation in the structure. The profiles of Na, Mg and CO_3_^2−^ are growing, with meaningful acceleration of growth in the regions close to the DEJ. The drop in the Young modulus occurs in the same location. It covers some 20% of the enamel width, close to the DEJ. It is worth noting that close to the DEJ, the numbers of the atoms of the latter ions are much higher than the number of Ca ions included in the “overbuilt structure”. It testifies to meaningful substitution of Ca by Mg, Na and K in the “overbuilt structure” in this location. Figure 4b shows the drop in Cl and F atoms per one Ca atom. There is striking parallelism of the curves, with significant quantitative surplus of Cl ions and real vanishing of F ions in one third of the enamel distance, starting from the surface with air.

It is a common opinion that fluorine involvement is responsible for the hardness of apatite matter. Figure 4c shows the comparison between the absolute concentration of F, expressed in percentage, and some excess of the hardness, observed close to the boundary of the enamel with the air. This additional excess is presented as the difference between the prolongation of the “normal” profile of the hardness, observed for the locations close to the DEJ and the real value of hardness at each point. The figure confirms the popular opinion.

## 3. Discussion

All the observed profiles of elements and chemical groups along the enamel width were not observed at a constant level. They changed in decreasing (Ca, P, Cl, F) or increasing (Mg, Na, K, CO_3_^2−^) modes. What is even more important, the profiles of the change were strictly exponential and our approximation of the variability of changes with exponential functions gave quite impressive correlation coefficients.

The approximation by using the simple exponential functions attracts our attention directly to the kinetic equations if one transforms the length axis into the time axis. It is reasonable to use the simple assumptions that the reaction front progress inside enamel is dependent directly on time progress and that the enamel width growth is also directly proportional to the time. Then, it is possible to imagine that two contradictory fluxes are going into the enamel: Mg, Na and carbonate supplies are going from dentin through the DEJ zone, while the opposite fluxes of Ca, P, Cl and F evolve from the enamel surface, supplied probably by mineral contents of saliva. Please note that the fluxes concern the excessive amounts of the ions mentioned and not the whole amounts. It is worth noting that Na and Cl fluxes are totally separated and roughly equal, although one should expect NaCl as the common source of both ions. The total reaction is as follows, going from the DEJ side to the enamel surface:

Ca_8.856_Mg_.147_Na_.401_(PO_4_)_5.658_(CO_3_)_.662_(OH)_?_Cl_0.027_F_0_ + 0.078F^−^ + 0.132Cl^−^ ⇔ Ca_8.856_Mg_0.022_Na_.143_(PO_4_)_5.713_(CO_3_)_0.284_(OH)_?_Cl_0.159_F_0.078_ + 0.125Mg^2+^ + 0.268Na^+^ + 0.378CO_3_^2−^ (1)

Here, we followed the results of calculations by Elliott [71] for the unit cell of the human enamel and adapted our results to his formulation and coefficients, taking strictly the same reference value only for Ca ions. Obviously, the notation above is only a formal one. In our calculations, we did not differentiate the PO_4_^3−^ and HPO_4_^2−^ species, and also the A and B substitutions by ion CO_3_^2−^ [72] In this approach, the averaged formula is:

Ca_8.856_Mg_.088_Na_.292_(PO_4_)_5.592_(CO_3_)_.457_(OH)_.412_Cl_.078_F_?_ (2)

**Figure 5:**
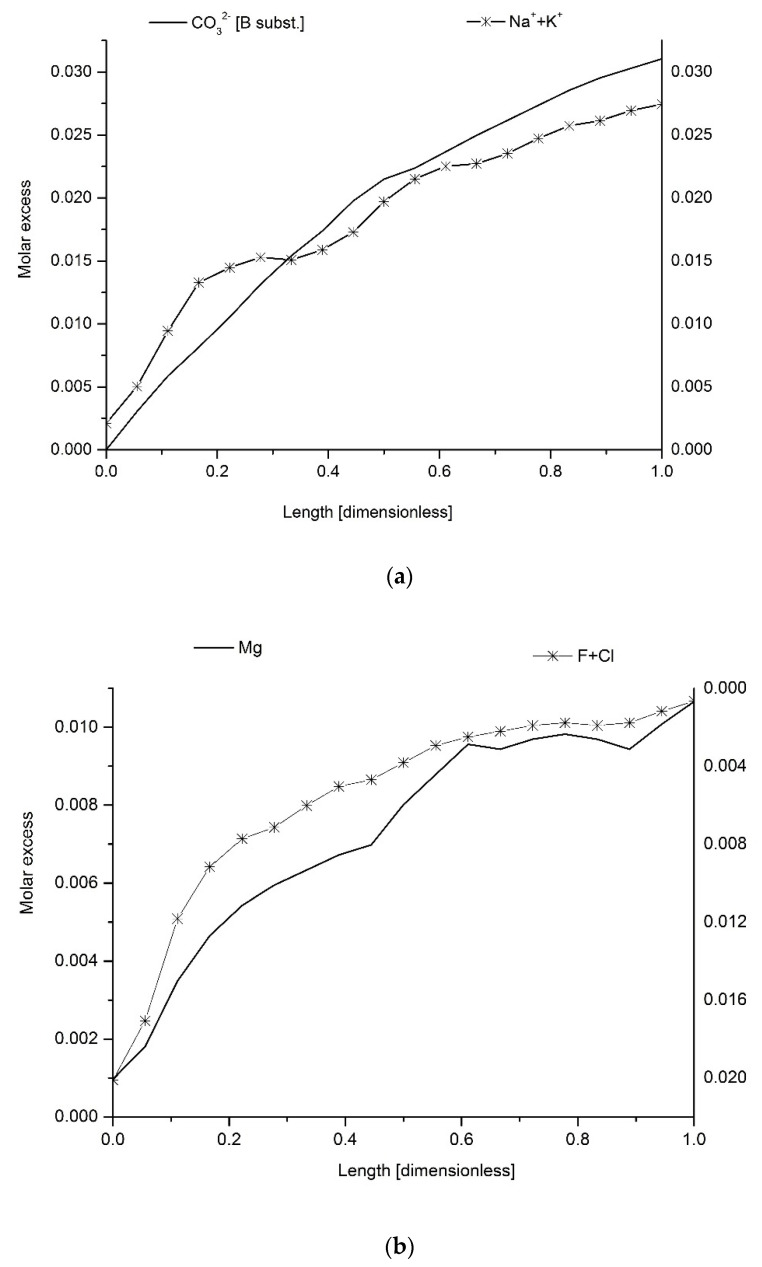

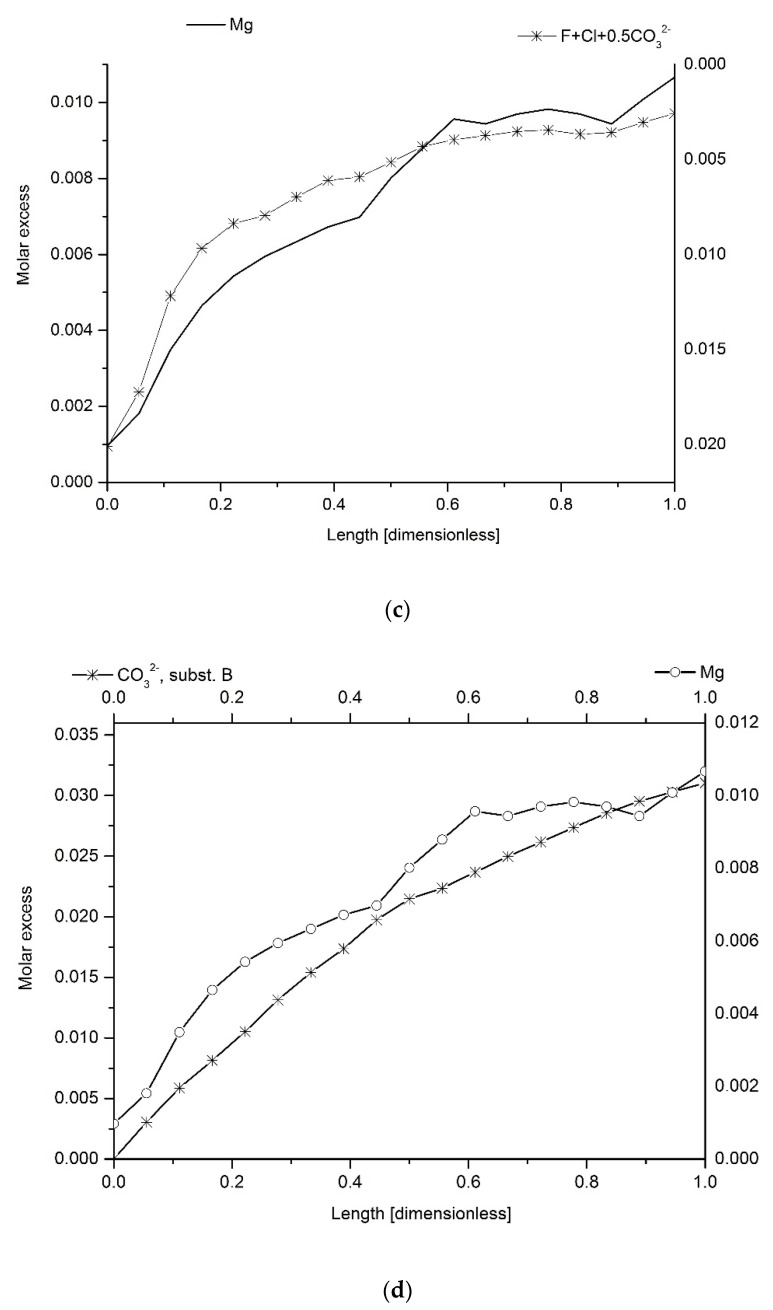

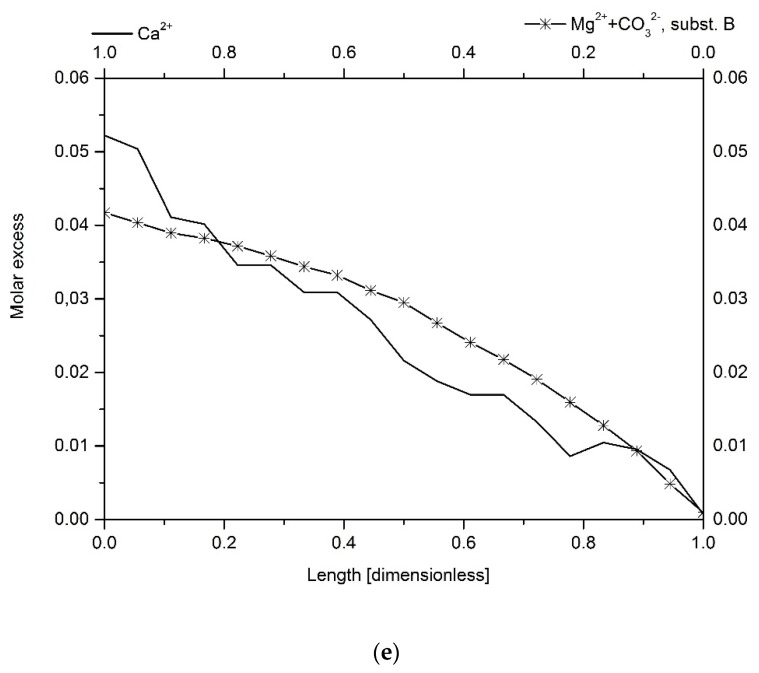
(**a**) Profile of supposed excessive CO_3_^2−^ distribution in substitution B along the enamel length compared with the summary profiles of ions Na and K, compensating the charge change; (**b**) profile of Mg distribution along the enamel length compared with sum of F and Cl concentrations. See the reversal of right y axes on (**a**,**b**), expressing the different directions of chemical reacTable 1. in (**a**), while 1:2 in (**b**,**c**) the same, but with correction involving the participation of CO_3_^2−^, substitution A; (**d**) comparison of Mg and CO_3_^2−^, subst. B ions, participating in different mechanisms, expressed by Equations (4) and (3), respectively; see the molar ratio 1:3; (**e**) balancing of real Ca loss against the profiles of 2 ions responsible for leaching Ca: Mg (Equation (4)) and CO_3_^2−^, subst. B (Equation (3)).

Some possible elemental and molecular exchanges are easy to imagine and to couple with one another. At first, we consider those exchanges which are connected with the rearrangement of electric charges. The exchanges of CO_3_^2−^ for PO_4_^3−^ (substitution B) must be most easily compensated with univalent cations, Na^+^ and K^+^ (Elliott [71])—see Figure 1a. This exchange was proved years ago by LeGeros. In Figure 5a, the profile of carbonates in substitution B (our measurements, with corrections calculated according to Elliott and confirmed by our Raman measurements, not shown here) is compared with the summary profile of Na^+^ and K^+^ ions. Taking into account the accuracy of the electron microprobe measurements for minor components and C, the agreement between the curves is very good. It contradicts the suppositions by Fleet and Liu [66] that the proportion of A and B substitutions can be close to 1:1 and confirms Elliott’s assumption of 9:1. Moreover, one can clearly see that the substitution is in stoichiometric ratio 1 CO_3_^2−^ (B):1 (Na^+^ + K^+^). Another substitution (A) of CO_3_^2−^ for OH^−^ should be compensated in the easiest way by the formation of an additional portion of HPO_4_^2−^ ions. Making a similar balance to that in Figure 5a is very difficult this time due to the excess of phosphates and difficulties in quantitative measurement of OH^−^ and HPO_4_^2−^ ions by Raman spectroscopy. The substitution in B location is treated here in the opposite way to the notion introduced by LeGeros and corresponds to the reaction below and means the attack on “carbonate” part of apatite by phosphates (Ca^2+^ ions are here the so-called Ca(2) ions) in a plane (001):

(3) 2Ca_3_(PO_4_)_2_*(Na,K)CaPO_4_*CaCO_3_*Ca(Cl,OH)_2_ + PO_4_^3−^ + Ca^2+^ ⇔ 3Ca_3_(PO_4_)_2_*Ca(Cl,OH)_2_ + CO_3_^2−^ + Na^+^,K^+^

The direction of this unit reaction from the DEJ towards the surface of enamel and the relevant formulae are introduced here especially to explain the distributions of elements. Freshly formed “overbuilt enamel” is of composition suggested at the first approximation at the left side of the equation. The flux of calcium and phosphate ions reacts and releases the equivalent amounts of carbonates and sodium and potassium. This type of reaction should result in an increase in parameter “a” of crystallographic cell towards the boundary with air. It is due to the dimensions of the ions (smaller CO_3_^2−^ and Na against greater PO_4_^3−^ and Ca) and the effect is steric.

Other exchanges are connected with other crystallographic sites. It is clear from Figure 1b that after elimination of carbonates and Na and K, as in Equation (3), remaining changes concern Cl (+ eventually F and CO_3_^2−^ (A)) and Mg ions. It is shown in Figure 5b, where the balance of exchange reaction is observed, in which involvement of Mg into the lattice excludes Cl and F from the apatite at the same time. Please note that this time, the direction of ordinate axes is inverted, in opposition to Figure 5a, where both components were parallelly involved in the lattice. In this case, the atomic ratio is 1 Mg:2 (Cl + F). The chemical exchange reaction is as follows, and formally means an attack on the Mg(OH)_2_ part of apatite roughly along the “c” (110) axis in the so-called hexad location:

3Ca_3_(PO_4_)_2_*Mg(OH)_2_ + Ca^2+^ + 2(F^−^,Cl^−^) ⇔ 3Ca_3_(PO_4_)_2_*Ca(F,Cl)_2_ + Mg^2+^ + 2(OH^−^) (4)

We have a proof from Figure 4a that reaction (4) is far-left-shifted for “overbuilt” enamel, in locations close to the DEJ. What is characteristic is that the course of the reaction leads to increasing total ionic character of some bonds, Ca^2+^ + Cl^−^ instead of Mg^2+^ + OH^−^, (chemical effect). One can somehow improve the accuracy of the relationship expressed in Figure 5b by adding the CO_3_^2−^ ions in substitution A (Figure 5c).

Figure 5a,b indicate two apparently independent attacks on apatite structure, presented in Equations (3) and (4). Figure 5d shows the close stoichiometric relationship between the reactions involved in mechanisms (3) and (4). The relationship is CO_3_^2−^(B):Mg = 3:1. Figure 5e indicates that the mechanisms are coupled, since the summary losses of calcium matter in the DEJ direction resulting from mechanism (3), which are equivalent to CO_3_^2−^(B) (corresponding to portion of Ca(2)) and from mechanism (4), which are equivalent to Mg^2+^ (corresponding to another portion of Ca(2)) nicely reach the measured Ca loss and they really sum up. After involvement of stoichiometric information, we can present the real coupled chemical, electrical and steric attack, which is expressed by the equation, leading from hypothetical phases to chlor(fluor)apatite:

3NaCaPO_4_*3CaCO_3_*Mg(OH)_2_ + 3PO_4_^3−^ + 4Ca^2+^ + 2Cl^−^ ⇔3Ca_3_(PO_4_)_2_*CaCl_2_ + 3CO_3_^2−^ + 3Na^+^ + Mg^2+^ + 2OH^−^ (5a)

or optionally:

2NaCaPO_4_*NaCa_2_PO_4_CO_3_*2CaCO_3_*Mg(OH)_2_ + 3PO_4_^3−^ + 4Ca^2+^ +2Cl^−^ ⇔ 3Ca_3_(PO_4_)_2_*CaCl_2_ + 3CO_3_^2−^ + 3Na^+^ + Mg^2+^ + 2OH^−^ (5b)

or:

NaCaPO_4_*2NaCa_2_PO_4_CO_3_*CaCO_3_*Mg(OH)_2_+ 3PO_4_^3−^ + 4Ca^2^ +2Cl^−^ ⇔ 3Ca_3_(PO_4_)_2_*CaCl_2_ + 3CO_3_^2−^ + 3Na^+^ + Mg^2+^ + 2OH^−^ (5c)

The left-side phase in Equation (5c) can be optionally expressed formally as Ca_3_(PO_4_)_2_*Na_3_PO_4_*3CaCO_3_*Mg(OH)_2_. Please, note that the mentioned phases show the same isomerisation as derivatives of tri-substituted benzene (1,3,5; 1,2,4; 1,2,3, respectively), due to the same symmetry in hexagon around the hexad axis. It is observed from the up/down perspective versus the hexagonal crystal, on the transverse cross-section. However, it is more complicated than for benzene since the isomeric location of CO_3_^2−^ and Na can be at different (0,0,1) levels (see Appendix A).

It is essential that PO_4_^3−^, Ca^2+^ and Cl^−^ and F^−^ act in parallel and exclude Mg^2+^, Na^+^ and CO_3_^2−^ ions. Taking the last equation into consideration, it seems that the maximum averaged admissible amount of Mg in enamel is close to 0.25%.

We introduced the formula for chlorapatite (optionally for fluorapatite) in the above equations since Figure 5b clearly indicates the line of the attack: Ca for Mg with the stoichiometric substitution of 2Cl^−^ and/or F^−^ and release of OH^−^ group instead.

**Figure 6:**
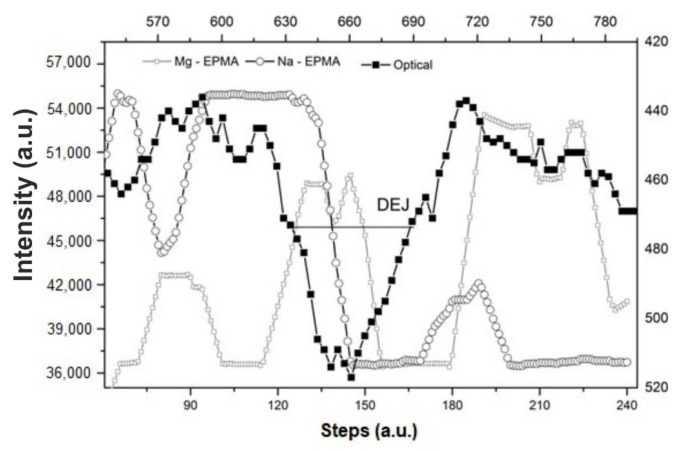
Distribution of Na, Mg and CO_3_^2−^ ions around DEJ. Enamel is on left side of the figure. Optical signal outlines the DEJ boundaries.

**Figure 7:**
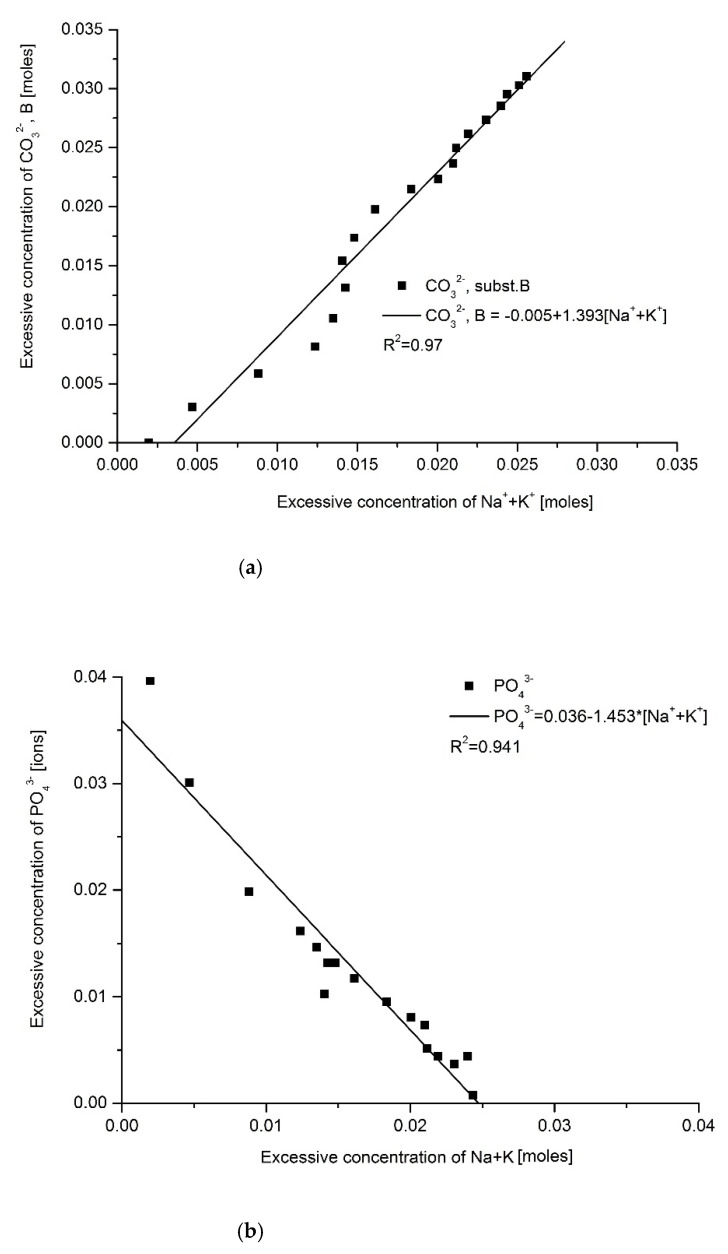

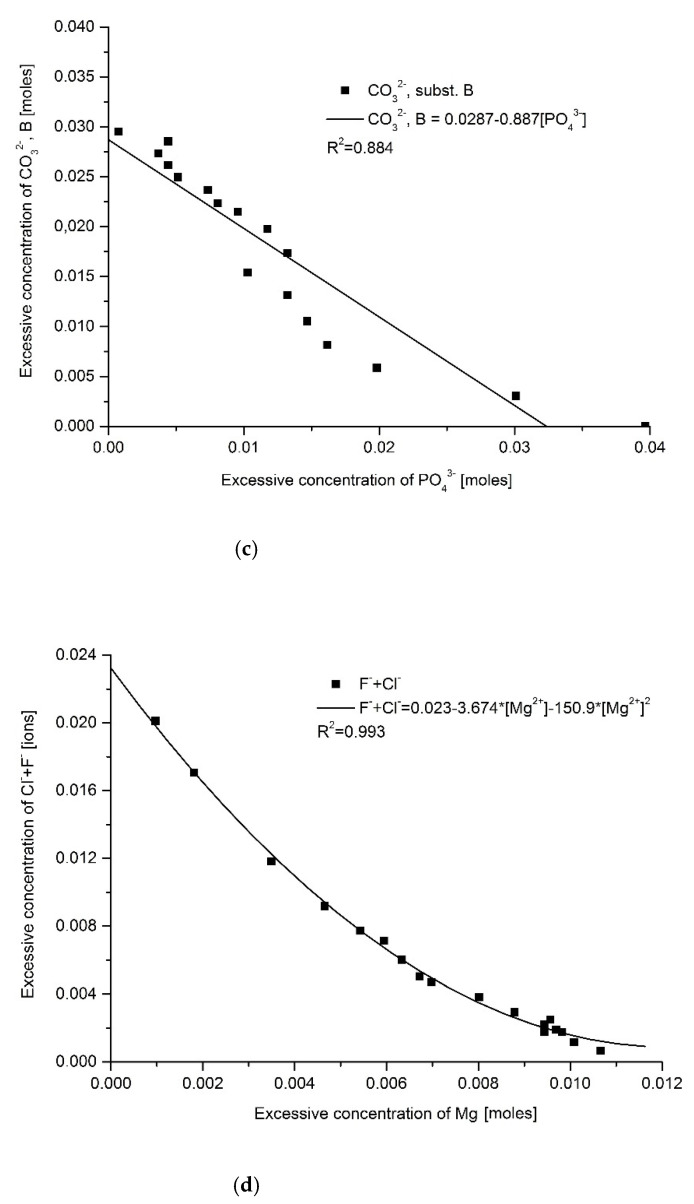

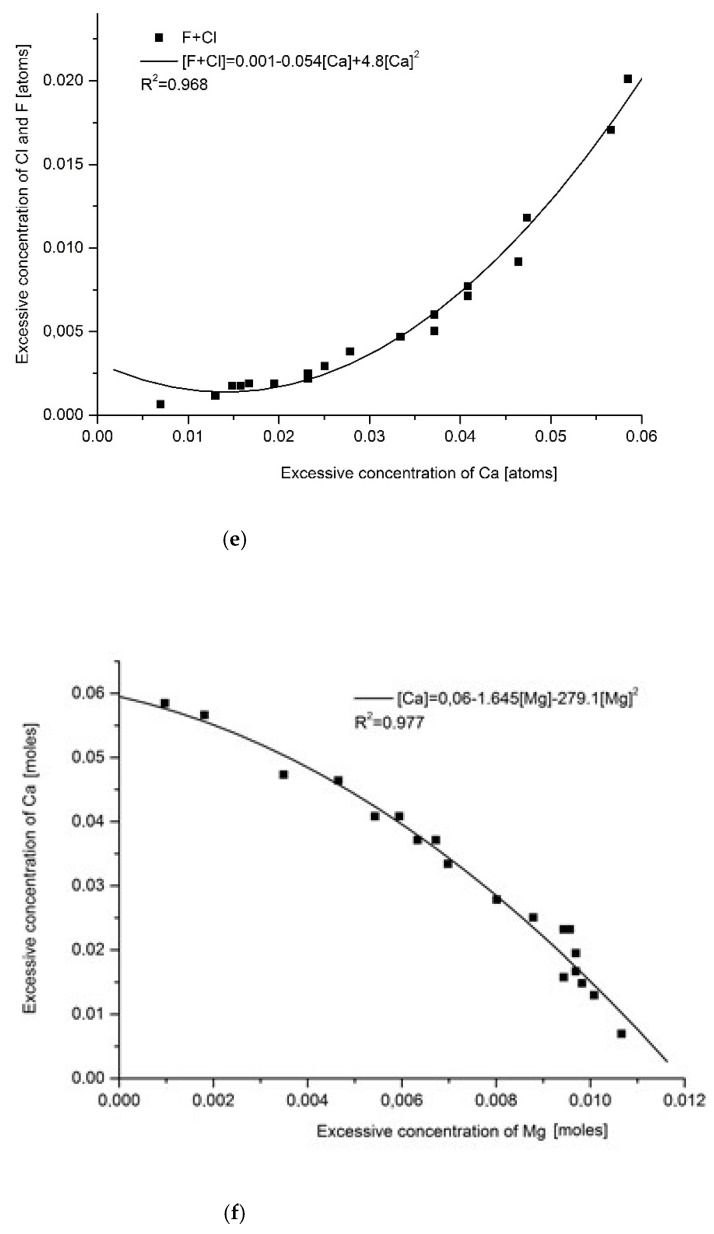

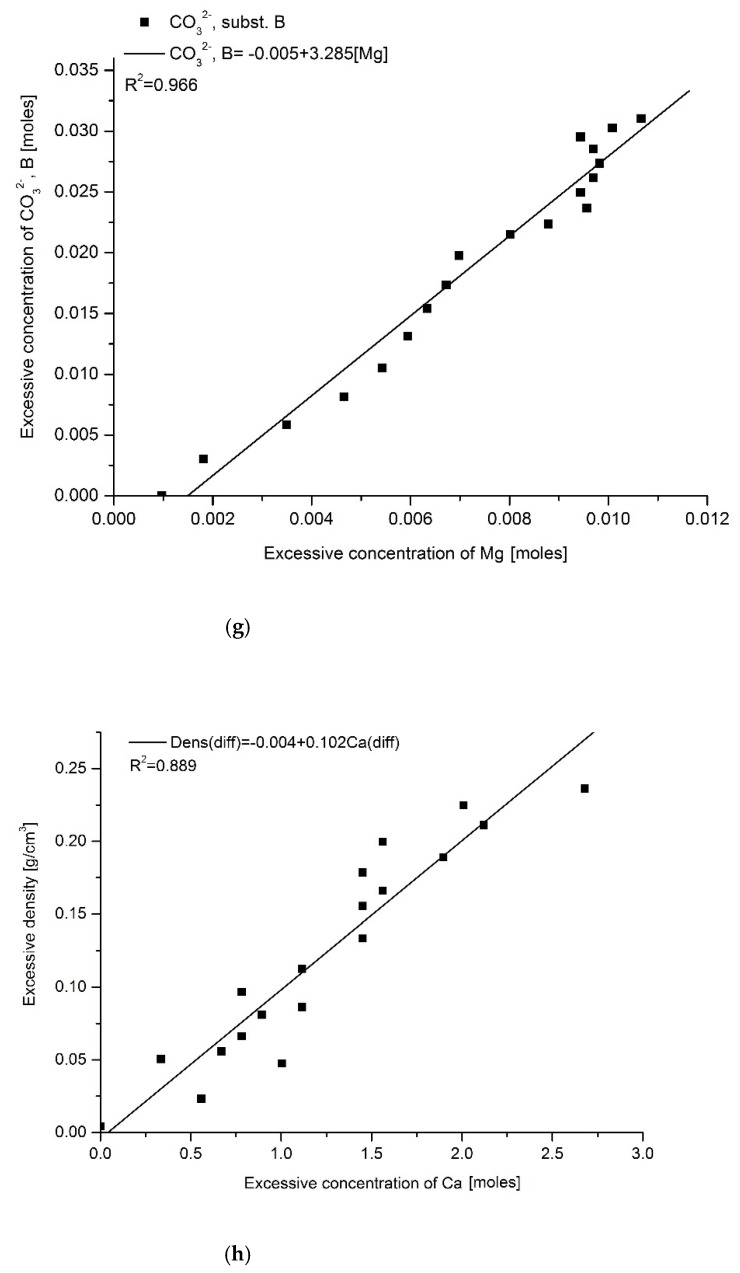
Correlations existing between the excessive amounts of some elements and other parameters: (**a**) CO_3_^2−^(B)—Na + K; (**b**) PO_4_^3−^—Na + K; (**c**) CO_3_^2−^(B)—PO_4_^3−^; all three relationships concerning Equation (3); (**d**) Cl + F—Mg; (**e**) Cl + F—Ca; (f) Ca—Mg; all three concerning Equation (4); (**g**) CO_3_^2−^(B)—Mg—concerning junction between Equations (3) and (4); (**h**) Ca-density.

Equation (5), expressing the coupled changes in the apatite crystallographic unit, is a complex one. Each time, it includes the action of nine ions on the three apatite type units. It is obvious that it cannot be performed in one moment (see also some nonlinear inter-elemental correlations in further Figure 7). Two separate mechanisms (Equations (3) and (4)) should act in some temporal order. Which reaction is an initial one? From the point of view of simplicity, it should be the reaction expressed by Equation (4), followed by three attacks according to mechanism (3) (inverted LeGeros mechanism). It seems reasonable that the attack starts at the key location—along the hexad axis. It is still another argument supporting this view. In our studies on the dentin enamel junction DEJ, Mg ions on the boundary of enamel–DEJ arrived inside of the DEJ zone, preceding Na ions and still preceding the Ca concentration jump (or optical outline of the DEJ–enamel boundary), characteristic of enamel (Figure 6). Thus, the removal of Mg ions joined with attack of Cl ions is the first step; it is followed by substitution of Na ions by Ca(2) ions, and this is followed by the substitution of CO_3_^2−^ ions by PO_4_^3−^ ones in inverted substitution of B-type.

Table 2 shows the above relationships in a more illustrative form, where all the atomic/molecular relationships are recalculated per 10^4^ atoms of Ca. It is easy to observe that the cited data by Wilson et al. [40], Aoba and Moreno [73], Hendricks and Hill [74] and Teruel et al. [75] concern the averaged values for enamel while the real parameters for minor species differ meaningfully on both ends of the enamel layer. On the other hand, the totally independent averaged data of the authors mentioned and ours show extraordinary convergence.

The intercorrelations between the particular elements are impressive if they are calculated between the excess values, i.e., belonging to the “overbuilt enamel”. Here, we grouped correlations as suggested in Equations (3) and (4). Figure 7a,b illustrate the influence of Na and K on anions CO_3_^2−^ (positively and linearly correlated; both kinds of ion cooperate in the operation of their release) and PO_4_^3−^ (inversely correlated; both ions compete; the anticorrelation is slightly asymmetric, probably due to influence of HPO_4_^3−^ ions). There is also an inverse and slightly asymmetric correlation between ions, CO_3_^2−^ and PO_4_^3−^ (competition) (Figure 7c).

The next three figures concern the Ca attack on Mg joined with the formation of chlorapatite. A nonlinear, second-order inverse correlation exists between Mg and Cl plus F; a positive and second-order correlation is observed for Ca and the sum of Cl and F—they are all introduced while Mg is released (Figure 7d,e, respectively). An inverse correlation of the second order is observed for Mg and Ca—the ions compete with each other (Figure 7f). The second-order correlation results here most probably from the fact that the attack of Ca and Cl/F on Mg and OH^−^ occurs in two stages. To check the coupling between reactions (3) and (4), we established a correlation between Mg (main attacked substance in Equation (4)) and CO_3_^2−^ (main attacked substance in Equation (3))—Figure 7g. The impressive directly proportional correlation indirectly, but in a very strong way, testifies to the fact that the actions expressed by Equations (3) and (4) are coupled. It is expressed by Equation (5).

As a supplement, the correlation between the excessive Ca content and the excessive density is shown (Figure 7h). Since we cannot expect any stoichiometric relationships in this case, the correlation is shown, in the simplest way, between the excessive density and the excessive percent concentration of Ca. Once more, the correlation is very clear. The same dependence was expressed earlier in similar form in Figure 3.

It seems that in layers somewhat deeper than the very boundary with the DEJ, a strictly controlled process of material changes starts in the overbuilt enamel, with gradual loss of some materials and gradual introduction of another one. We should remember that the convention assumed for figures in this paper is that the surface of the enamel with air is put always on the left side, and for that reason, we can just speak about the decaying stream of phosphates. If one considers that the time of passing of chemical reaction front can be treated as proportional to the width of enamel, then the curves shown in the figures transform into the kinetic curves for two inversely directed chemical reactions. The extremely successful use of exponential approximations for expressing the concentration profiles (Figure 2) is another element inclining to kinetic considerations. Such profiles of kinetic curves mean that the opposite reactions reach the equilibrium state (see the dotted lines in Figure 1a,b, showing the location around relative distance *x* = 0.3). The initial material composed of hydroxyapatite with small participation of chlorapatite, practically without fluorapatite and much enriched in carbonates, Mg and Na, transforms into the protective material composed of hydroxyapatite with admixtures of chlorapatite and fluorapatite, with relatively small additions of Na, Mg and CO_3_^2−^ ions. There is a concerted action of Ca, Cl and F ions against the Na, Mg and CO_3_^2−^ ions. The averaged concentration of elements in enamel as expressed, e.g., by Wilson et al. (see fifth column of Table 2), Aoba and Moreno (sixth column) and in our results (fourth column) deviates in two directions:

(1) Towards the enamel surface—to the mixture of three apatites—essentially, hydroxyapatite with chlor- and fluorapatite; this material is only faintly saturated with Na, carbonates and hardly with magnesium.
(2) To the DEJ—the ultimate result is the existence of the apparent phase expressed as 3NaCaPO_4_*3CaCO_3_*Mg(OH)_2_ or, optionally, Ca_3_(PO_4_)_2_*Na_3_PO_4_*3CaCO_3_*Mg(OH)_2_ in the “overbuilt” enamel; since the enamel is formed later than the dentin and from the dentin side, we can treat the Na^+^/Mg^2+^/CO_3_^2−^- rich layer as the original one in enamel and transforming itself into a layer mentioned in point 1.

One can assume the hypothesis expressed in Figure 1 that two opposite streams are passing through enamel from two ends and both overbuilt phases behave dynamically. This version can be enhanced by the arguments that post-mortem enamel is additionally fluorinated by similar supplies of external fluoride. We can consider, hypothetically, that the soft enamel is quickly formed, with maximum content of carbonates, Mg, Na and K ions going from the DEJ. This original phase is later attacked by streams of Ca, P, Cl and F ions. Then, the soft enamel is “stationary” and is attacked by a dynamic flow of the ions mentioned. However, in this case, it is difficult to speak about substitution B of carbonates in place of phosphate ions. There are original carbonate ions which are attacked and substituted by the phosphate ions during the maturation process. Alternatively, we can consider formation of the original phase, with composition strictly to the averaged one described in the fourth column of Table 2, self-segregating later in two directions into extreme phases.

We treat enamel from the formal point of view as a structure consisting of two phases—the “core” phase, expressed in Table 1, and the “overbuilt” phase split into two components: the component of mixed apatites with serious contribution of fluor- and chlorapatites close to the surface and the component approaching extreme formulae 3NaCa(PO_4_)*3CaCO_3_*Mg(OH)_2_ close to the DEJ. Both components in the “overbuilt” enamel behave as a mutual solid solution, passing in a continuous way to the extreme compositions. We do not know if the component 3NaCa(PO_4_)*3CaCO_3_*Mg(OH)_2_ really exists in a crystallographic sense, but the enrichment of enamel in Mg and Na and loss of Ca and P on the boundary with the DEJ are real facts. Such Mg and Na ions must be kept somewhere in a solid structure. This imagination of phases is here simpler than the 4-phase structure proposed by Driessens and Verbeeck [77]. Although their phases seem to be well known in crystallography, it is not sure if some of them can really coexist in enamel, e.g., dolomite.

It seems that although one can consider the mechanisms described by Equations (3) and (4) as separate ones, there is indirect proof that the mechanisms are coupled at the level of crystallographic units:

(a) The molar relationships are close to stoichiometric not only for each mechanism on its own but also between species participating in different mechanisms (see Figure 5c);
(b) The correlation coefficients between the species participating solely in mechanisms (3) and (4) are very high (see Figure 7g);
(c) The equilibrium points for elemental curves for both mechanisms, shown by the arrows, are localized very close (Figure 1);
(d) There is a specific stoichiometric relationship between Mg and Ca species, corresponding to the formal formula 3Ca_3_(PO_4_)_2_*Mg(OH)_2_.

What is worth noting is that the stoichiometric relationships are as follows: 3 CO_3_^2−^:3 Ca(2):3 (Na + K):1 Mg:1 Ca(2):2 (Cl + F) (see Table 3). It leads to the connotation that the apatite crystal behaves according to its formal stoichiometric formula; three Ca_3_(PO_4_)_2_ are formed by entering of phosphates in locations of carbonate groups in each unit and it is associated with one substitution of one Mg by Ca(2) and introduction of two Cl or F ions in a Ca(F,Cl)_2_ formal subunit. Then, the real equation of the coupled reaction at the level of unit cells can be expressed in a form of Equation (5).

### Energetic Relationships

It is interesting to invoke to the crystallographic data given by Al-Jawad et al. [69]. One can consider energy difference resulting from reaction (3) at particle level. We use a specific form of Braggs law:

n*12.4/E = 2d*sinΘ (6a)

but for (300) line used for calculation “a” for hexagonal structure and for *n* = 1, we have for values of angles which are slightly different in two locations:

21.477/(a_1_sinΘ_1_) = E_1_ (6b)

and

21.477/(a_2_sinΘ_2_) = E_2_ (6c)

Since the energy of exciting radiation is constant, we can ask what happens when we put value of a_1_ into Equation (6c). In fact, one seeks the energy shift in exciting radiation which will allow fulfilling Braggs law. Otherwise, it corresponds in a trivial way to the energy which is connected with going on reaction (3) and transformation in plane (001).

ΔE = 21.477/a_1_(1/sinΘ_1_ − sinΘ_2_) (6d’)

Or alternatively:

ΔE = 21.477/sinΘ_1_(1/a_1_ − 1/a_2_) (6d’’)

**Figure 8:**
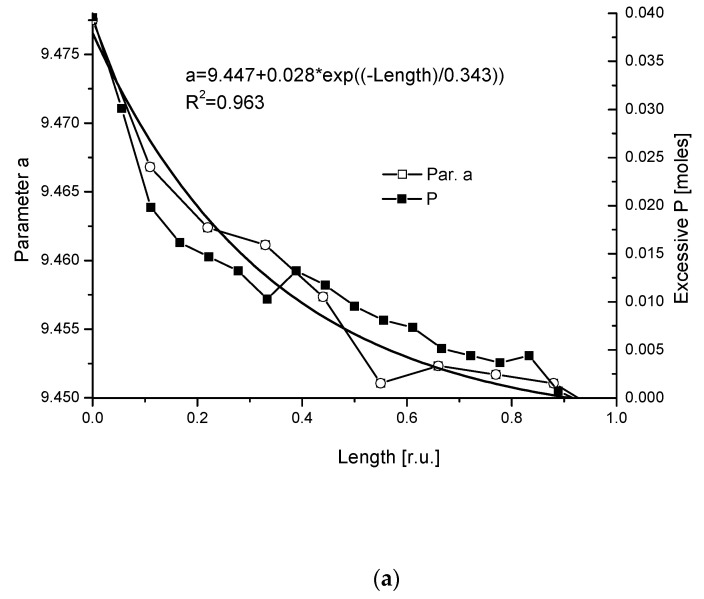

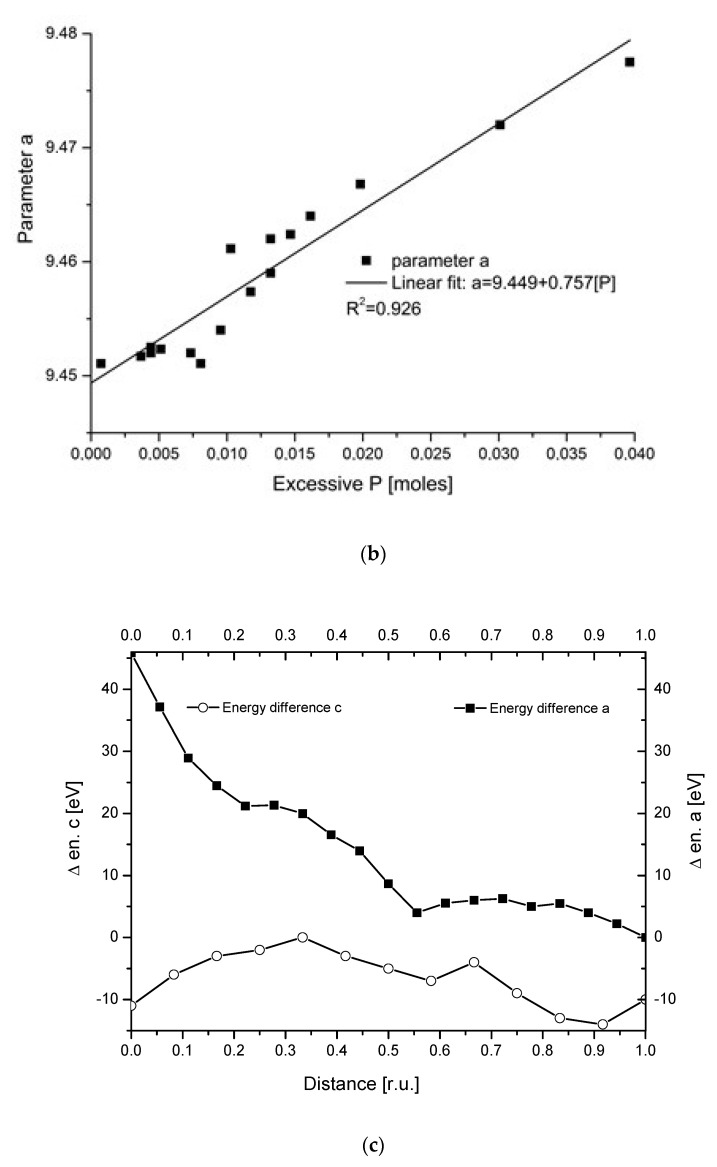
(**a**) Profile of variability of crystallographic parameter “a” (black line with open squares) with its exponential approximation (solid line) as compared with profile of P (black line with solid squares); (**b**) correlation between parameter “a” and excessive P contents; (**c**) changes in energy of transformation in plane (001) and perpendicularly to it.

After calculations, using angular values from microdiffraction measurements, the energy is equal to 46 eV, going from DEJ to boundary of enamel with air. The distribution of energy along the route from the boundary with air to the DEJ obeys exponential relationship and resembles the distribution of phosphates, which seems to be reasonable (Figure 8a,b). One can assume that this energy increase results from the energy necessary for atom rearrangement in crystallographic network. Of course, some part of the energy can be ascribed to the mechanical stress in teeth, but we suppose that this component is small. The structure of apatite in vicinity of contact with air is more ordered than of this one at the boundary with DEJ and the transformation is antientropic.

The same can be repeated for line (002), with coefficients 12.4 in Equations (Ib) and (Ic), and the c parameter instead of the a one. This time, the energetic difference between initial and final states as taken from Al-Jawad et al. [69] is minimal, ~1 eV, but intermediate states have energies lowered by up to 14 eV (Figure 8c). Passing the tetrad channel (mechanism (4)) is much easier than moving in plane (001). It is confirmed in an indirect way by independent data on diffusion in apatites. The argument results from the data given by Royce [83]. He indicates that the ionic jumps along dimension “a”, especially intercell movements, demand the energy of 10 eV per dislocation while the transport along hexad axis demands only 0.5 eV.

The same calculated for parameter d from Bragg’s rule gives 45.8 eV and it corresponds to energy of total rearrangements as expressed by Equation (5). Simple recalculation informs us that it corresponds to the value of 4420 kJ/mol, where moles means the moles of “overbuilt enamel”. The reactions concern the “overbuilt” enamel only. The averaged formula for enamel apatites gives the mass equal to 926 g, while only 6.5% of this corresponds to “overbuilt” enamel, so we should consider the mass 926*(100/6.5) = 15,433 g. We can estimate the volume of enamel in average molar teeth as 0.1 cm^3^; thus, the mass is ~0.3 g, and we found that a mole of “overbuilt” enamel split among 51,400 molar teeth. The energy of transformation of enamel in a single molar tooth is equal to 86 J.

Finally, we can invoke the data from Table 1 to check some of our assumptions. For example, from the data referring to density, we can see that the density of enamel close to the DEJ is increased by a coefficient of 1.0449 if we go to enamel in vicinity of the boundary with air. A parallel mass change resulting from variable composition of apatite is equal to 1.0396. Introduction of volume correction resulting from the change in crystallographic parameter “a” equal to 1/(a_air_/a_DEJ_)^2^ gives a small correction, leading to a value of 1.0333. Parameter “c” was not considered since it is nearly the same in extreme points. It is clear that the change in density resulted mainly from the change in composition and it confirmed our assumptions. The change in crystallographic cell volume had only secondary meaning.

During recent years, several contributions were published where the authors described the nanocomposition of enamel crystallites in rodent [84,85] and human teeth [86,87]. They established very great importance of fluorine, sodium, magnesium and carbonate ions at crystallite level. Our studies concerned many ions, including those mentioned above, at crystal level and at macroscale, on a linear cross-section along the enamel. The role of future investigations is to correlate those two approaches.

## 4. Materials and Methods

Samples of mature molar teeth were collected from healthy patients and additional ones were taken from patients in accidents. There were 10 samples in the first class and 8 in the other one. All were collected from adults in the age range of 20–35 years, with fully shaped and sound teeth. The samples were cut transversely into thin slices along the long axis (perpendicularly to the buccal and lingual surfaces) of the tooth with the slow diamond saw supported with air cooling. After the first cut, the cross-sectioned surface was very carefully polished in the end with silica powder. The surface roughness was finally established in approximation of 1 µm to fulfill the demands imposed on the quality of surfaces analyzed by the SEM method. Then, the thin (~0.25 mm) slices were cut off from the rest of the tooth—5 items from the first class mentioned above and 4 from other one. The rest of the tooth was treated as a reserve. The slices were kept in slightly (0.1 M of Sorenson P5244 buffer) treated water for a short time and they were taken off from the water and carefully dried before the measurement.

The scanning electron microscope VEGA LMU (manufactured by Tescan, Brno, Czech Republic) equipped with an INCA Energy 450 VP unit with X-Act Premium energy dispersive detector (Oxford Instruments, Abingdon-on-Thames, UK) was used for making elemental profiles in direction: tooth external surface—dentin enamel junction (DEJ). The settings were established as follows: accelerating voltage at 20 kV and beam current at 0.7 nA. The excitation was optimized on detection of elements between sodium and calcium. The energy resolution of the detector was conventionally checked on the MnKα line and was equal to 130 eV. To detect C (assumed to be mainly carbonaceous origin, except the 15-µm wide increment touching the DEJ zone), the measurements were performed on the polished surface without any deposition. The unpolished surface of the thin slice was put on the conductive glue to avoid charge loading. The corresponding backscattered electron images of the area of interest were stored.

In an independent way, images from the optical microscope were collected. The Eclipse E400 optical microscope was applied for the observation of the samples, especially in the measurement regions. A Coolpix 950 digital camera (Nikon Europe B.V., Amsterdam, The Netherlands) was used for taking photos. The images of interest were stored in the digital form for comparison with the chemical and mechanical results. The analysis of the images was performed using the image-processing program Micro Image 4.0 by Media Cybernetics (Rockville, MD, USA). Linear profiles were extracted from the images for comparing the optical look with the chemical profiles [88]. The quantification was performed using the PAP procedure [89], taking pure hydroxyapatite samples as a standard sample. Due to the known difficulties with the excitation of F, the estimated concentrations of that element could be lowered in comparison with the real values. The same concerned the well-known difficulties in determination of C [90]. Nevertheless, the total trends in profiles of both elements were clearly observed and were very similar to the trends known from the literature.

Phase analysis of the chemical structure of teeth was carried out with the use of a tabletop XRD device—Philips X’Pert, applying a Cu tube. The applied voltage was set as 35 KV, and current at 0.8 nA. The linear X’Celerator detector was selected. The X-ray bundle was squeezed with a capillary of 50-µm diameter. The sample was shifted before the opening of capillary with 50-µm long steps. The collected data were compared with the JCPDF (International Center for Diffraction Data) database. The DIFFRAC plus software ensured the automatic comparison and identification of the diffraction spectra; alternatively, the comparison was made with the Match! software (by CRYSTAL IMPACT Dr. H. Putz & Dr. K. Brandenburg GbR Bonn, Germany). 

The dispersive Raman spectrometer inVia Reflex (by Renishaw, Wotton-under-Edge, UK) was used for Raman line determination. A Leica stereoscopy microscope was attached for the observation of locations on samples. The charge-couple detector (CCD) detector was cooled with the use of liquid nitrogen. The sample excitation was made with laser diode emitting radiation of 785 nm. The applied power of the laser was equal to 300 mW. One action of the laser irradiated a spot of ~1 µm diameter. The spectral range was selected from 400 to 3600 cm^−1^, to detect all potentially significant lines of both organic and, where possible, inorganic character. The spectral resolution was about 2 cm^−1^. In this contribution, a Raman microscope was mainly used for the determination of carbonate bands, with special attention towards lines corresponding to B substitution of carbonate.

Due to the random character of individual results and to the need of generalization when it concerns the total shape and mutual relationships in chemical results, the majority of results were recalculated into dimensionless relative units, i.e., the total width of enamel along the scan path was always recognized as one unit length and all the absolute locations inside the sample were related to the total width of enamel. The position at the enamel surface was assumed to be 0 and the location of the contact with the enamel–dentin DEJ was 1. In the same way, where necessary, the ordinate parameters were compared with the maximum value of a given parameter and transformed into relative values. Such a system of data presentation enables comparisons with other results, independently of the widely different individual cases and methods.

Our paper is arranged in a way to present, at first, our own quantitative results and concepts in the Results section. In the discussion section, the results of other authors which are compatible with our concepts are presented and compared and some more sophisticated calculations concerning XRD results are added. The main aim is to present comprehensive quantitative imagination of enamel.

## 5. Conclusions

The careful estimation of chemical profiles of different elements in enamel with superposed micromechanical and other data adapted from other authors revealed the essential features of enamel. From a formal point of view, enamel can be divided into the “core enamel”, which is an essential and stable structural element of the whole enamel zone, and the “superstructure”, overbuilt directionally on the “core”, involving some 6.5% of the whole enamel mass. Concentrations of elements such as Ca, P and Cl drop regularly and in an exponential way from the surface of enamel towards the boundary with the DEJ. Opposing this, the concentrations of Mg, Na, K and CO_3_^2−^ increase in an exponential way in the same direction. Fluoride ions are an exception. The concentration of the latter element drops in an exceptionally quick way and it vanishes to negligible values in a distance about 1/3 of the whole dimensionless enamel thickness starting from the boundary with air. It is in accordance with the assumptions about mobility of F and OH ions in apatite lattice [91]. Profiled concentrations of all the mentioned elements are coupled with one another in either a directly or inversely proportional way, always in a nearly strict stoichiometric way. The vanishing Mg, Na and CO_3_^2−^ ions make place for Ca, PO_4_^3−^, Cl and F. The concept of “overbuilt” enamel is quite original and efficient in explaining tooth chemistry. The quantitative coupling of chemical and physical features confirms the validity of the concept. Similarly, our original method of calculation of energy of chemical transformations provides a good tool for searching the energetics of enamel.

One should keep in mind that although we present the phenomena of internal spatial changes inside tooth as deterministic reactions, they are, in reality, probabilistic processes. We have noticed many points leading to only probabilistic relationships. For example, the proportion of substitutions of CO_3_^2−^ in locations A and B is only determined by Elliott as a constant; really, it is not studied here more deeply, although the application of Elliot’s approach brought good results. In the same way, the proportion between the substitutions by Na and K is better known, but we are not sure if it is a universal relationship, especially when taking dietary habits into account. The lattice defects arriving during these reactions are known but their spatial distribution is not strictly determined. In addition, all the chemical concentration estimations from electron probe microanalyzer (EPMA) are imposed with methodical and statistical errors. Nevertheless, the huge number of EPMA results gives the impression of nearly ideal regularity. In light of such constrai(nts, the results given in our contribution seem to be extremely regular.

We must recognize that the presence of Mg, Na, K and CO_3_^2−^ weakens enamel and makes it less perfectly crystallized [92]. It seems that some forms of the carries can occur in a similar way as the reaction expressed in Equation (5), but are not stopped in due time. The proof can be found in the composition of the walls of carries’ openings. In many studies, and ours also (not shown here), at locations close to the openings, elevated amounts of Mg, Na and K are detected.

Finally, although tooth enamels seem to be wildly different, after normalization of units, they become surprisingly regular and repeatable. The presented results open a new way for the structure design of nanocomposites for possible biomedical application, such as dentistry, prosthesis, bone and tissue engineering.

## Figures and Tables

**Table 1:** Basic chemical values of the enamel core and mechanical data.

**Element/ion**	**Ca**	**P**	**Na**	**Mg**	**Cl**	**F**	**CO_3_^2−^**	**K**
Amount	35.63%	17.12%	0.35%	0.057%	0.094%	~0	1.8%	0.07%
Relative concentration	0.944	0.916	0.387	0.162	0.158	0	0.462	0.727
**Parameter**	**Hardness**		**Elasticity** **Modulus**			**Density**		
Value	3.55 GPa		79 GPa			2.887 g/cm^3^		
Relative value	0.822		0.918			0.957		

**Table 2:** Elemental content of the enamel compared with data according Wilson et al. [40], Aoba and Moreno [73], Driessens [26], Hendricks and Hill [74], Teruel et al. [75], Combes et al. [76].

Element	Surface Location	Location Close to DEJ	Mean Values	[40]	[76]	[26]	[73]	[75]	[74]
Ca	10,000	10,000	10,000	10,000	10,000	10,000	10,000	10,000	10,000
P	6451	6389	6420	6314	6317	5991	5981	6227	6136
Mg	25	165	95	99			190	137	114
Na	162	453	308	330		282	116	405	
CO_3_^2−^	321	748	535	516	540	509	475	759	568
Cl	179	30	105	88		65		103	
F	88		7			11			

Mean values for all measurements: P = 6247; Mg = 118; Na = 294; CO_3_^2−^ = 552; Cl = 95; F = 35.

**Table 3:** Stoichiometric relationships in “overbuilt” enamel, compared to other known stoichiometric ratios in enamel (with recalculations of coefficients bringing them closer to our ones are given in brackets).

Author	Ca	Na	PO_4_^3−^	CO_3_^2−^	Mg	Ca	Cl
This paper	4	3	3	3	1	1	2
Driessens [26]	1 (3)	1 (3)	1 (3)	1 (3)			
Driessens [78]	3	2	3	3			1 (OH^−^)
El Feki [79]	2	1	3	3			
Chickerur [80]	2 (3)	1 (1.5)	1 (1.5)	1 (1.5)			
Bonel [81]	1 (3)		1 (3)	1 (3)			1 (3)
Bonel [81]	3	2	3	3			1
Le Geros [82]	2 (3)	2 (3)	1 (1.5)	1 (1.5)			1 (OH^−^) (1.5)

**Figure A1:**
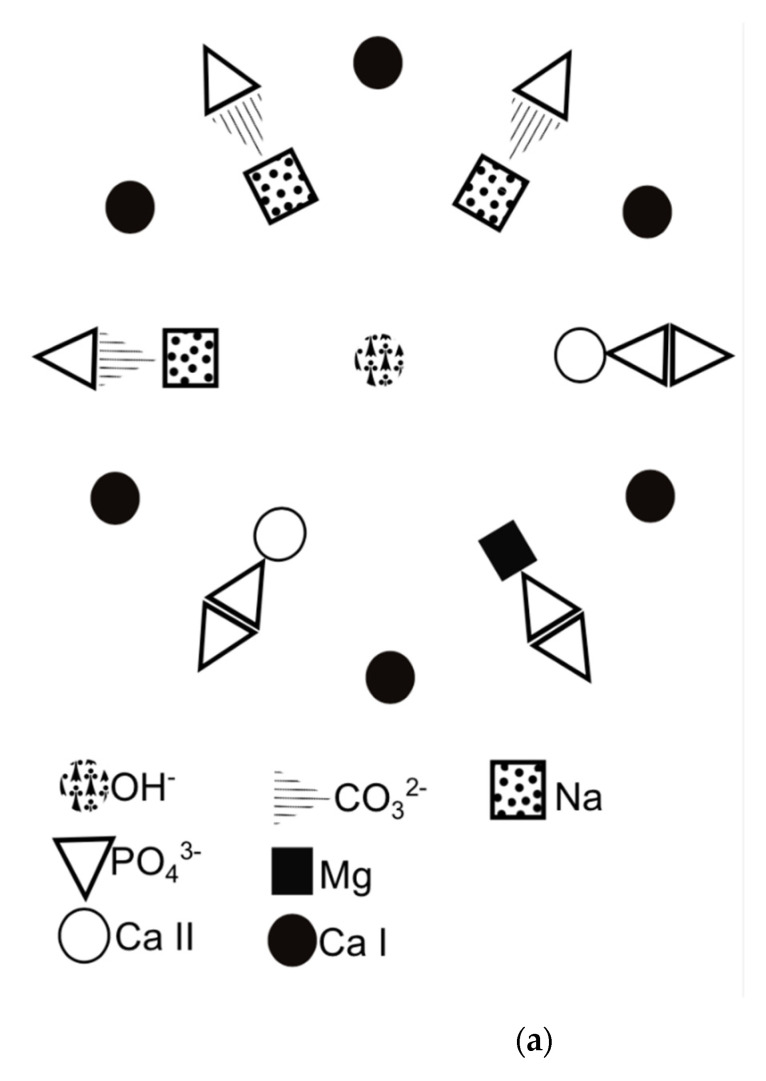

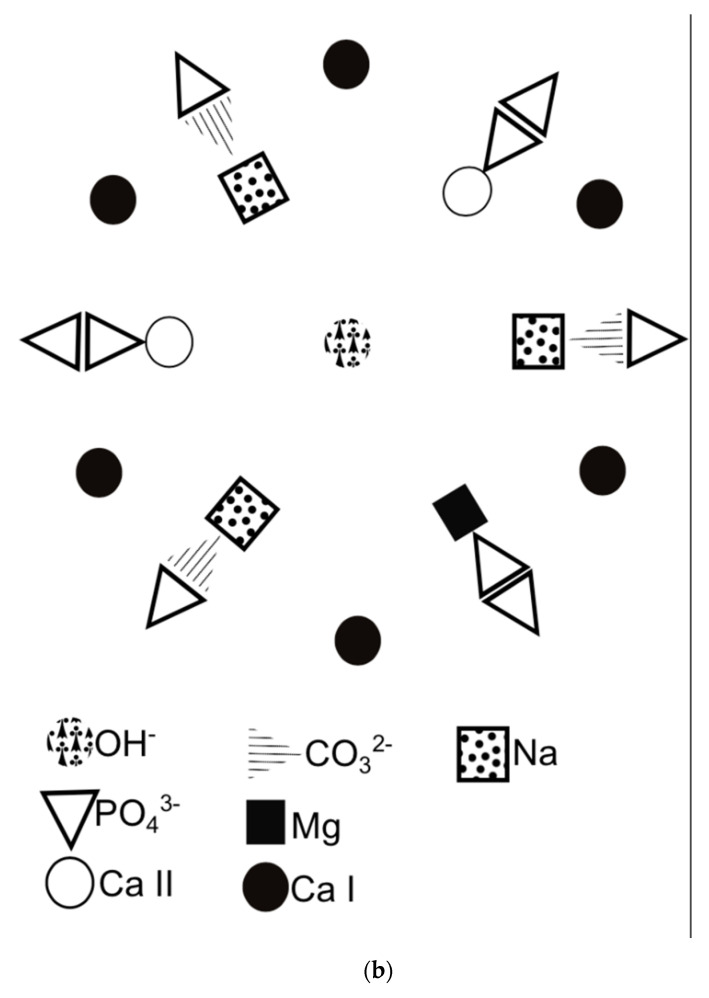

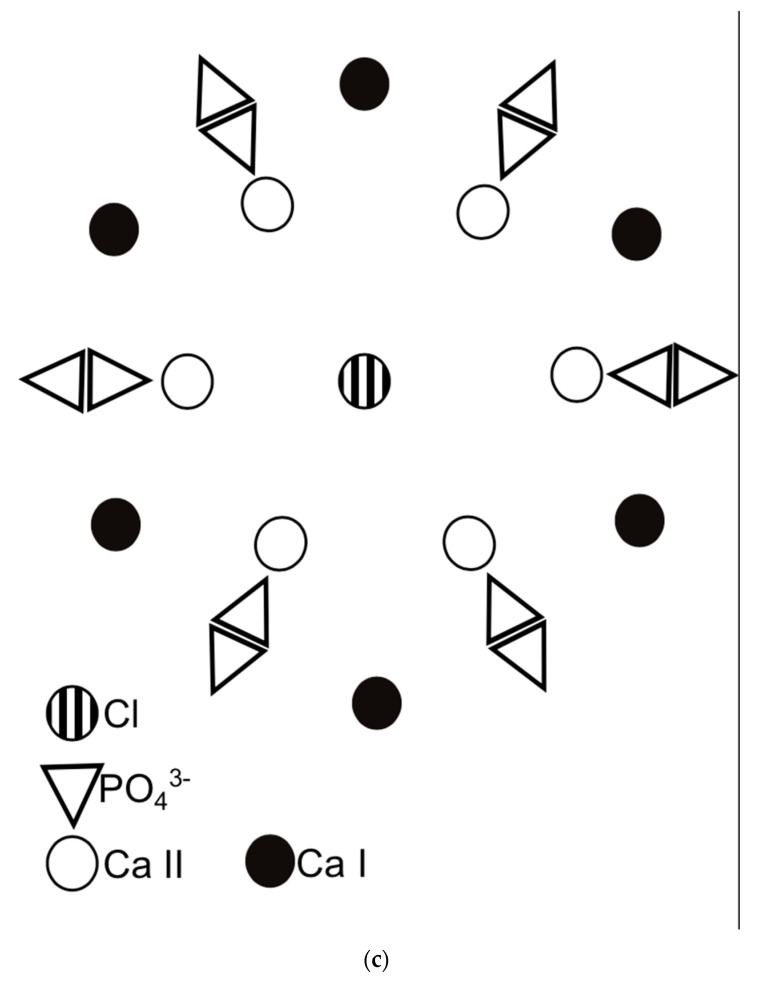
(**a**) Model of the crystal of “overbuilt” apatite in position close to DEJ, centered around hexad axis; Na and CO_3_^2−^ ions are symmetrically located, while Mg in asymmetric way; OH^−^ ion is present. (**b**) asymmetric version; (**c**) on the boundary enamel-air, all Ca(2) ions and phosphates are present, Cl entered the cell.

## Data Availability

Data sharing is not applicable to this article.

## Appendix A

The illustrative imagination of the changes going in the “overbuilt” enamel according to routes given in Equations (3)–(5) is suggested. Figure A1a shows the rearrangements going on in the unit cell, close to DEJ. In the Ca(II) locations, three neighboring Ca ions are replaced with the Na ions. This configuration is highly asymmetric and probably unstable from chemical point of view. In one of Ca(II) ion triangles, one position is overtaken by Mg ion, which means even greater perturbation in symmetry. Another extreme configuration is possible as well. Around, each second Ca(II) calcium is replaced with Na ions and each second phosphate location is changed with the carbonate ions. This configuration is symmetric (Figure A1b), except Mg involvement.

All it means, in summary, that the wide hexagon around hexad axis was shrunk in plane (001) in comparison with traditional particle of hydroxyapatite. Moreover, how it is known from the paper by El Feki et al. [79], the increasing presence of Na and/or CO_3_^2−^ ions forces OH^−^ ions to increase the distance from plane z = 0.25, which is the location of Ca(II) triangle.

In contrast, Figure A1c characterizes the close to the surface fragment of the unit cell, with all Ca(2) ions present and Cl or F instead of OH^−^ ion.

If one observes the transverse cross-section of the hexagonal structure from the top, the benzene-like isomers are characteristic of this arrangement. The configuration is disturbed by the presence of Mg ion among the Ca(II) ions in the triangles of Ca(II) ions.

One can imagine a version of figures, in which Mg ion partially occupies (CaI) locations.
